# Advancing Big Ideas with *Small Science*

**DOI:** 10.1002/smsc.202000068

**Published:** 2021-01-04

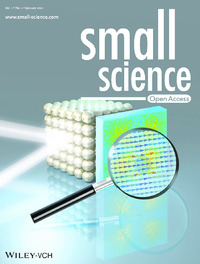

We are very proud to present the first issue of *Small Science*, Wiley's new premium open access journal for research at the sub‐macroscopic level. Proverbially, big ideas start with small steps and, in the context of scientific research, we hope that your big ideas will start with *Small Science*. With the highest production standards as well as a fast, fair, and selective peer‐review process, this journal is the perfect platform for making your big ideas and your best research available to the whole community.

The launch of our journal takes place right at a time when the COVID‐19 pandemic has had a major impact on our society. Extensive and strict shutdown measures, essential to prevent the spread of the disease, have largely restricted regular human activities. While medical professionals fight COVID‐19 from the front line, scientists and researchers are working diligently on the development of vaccines, testing methods, and treatments. Tackling tiny with tiny—nano‐based solutions are being prepared to fight the invisible enemy in a wide range of fields as diverse as biology, medicine, engineering, chemistry, materials science, and computational science. From this perspective, the pandemic highlights the interdependence of the challenges our society is facing and the vital role of scientific and technological progress: fast global exchange and easy accessibility of scientific results have never been more important.

Accordingly, our new premium journal not only aims to publish your most innovative and impactful research as open access; *Small Science* will publish accepted, unedited articles as soon as possible. The final, edited version will replace the submitted version once it is ready. This way, your work is made accessible to everyone, faster–globally, freely, and immediately.

Your manuscripts are handled most professionally by a team of highly experienced and independent in‐house editors who strive to make *Small Science* your journal of choice for top‐quality research, publishing Research Articles and Reviews in addition to Perspectives and Opinions.

Ten highly renowned and prominent voices in the nanoscience community have been appointed as Executive Advisory Board Members:

**Khalil Amine**, *Argonne National Laboratory, Lemont, IL, USA***Gero Decher**, *Université de Strasbourg, France***Ulrike Diebold**, *Technische Universität Wien, Austria***Harald Fuchs***, Universität Münster, Germany***Hideo Hosono**, *Tokyo Institute of Technology, Japan***Sumio Iijima**, *NEC Corporation, Tsukuba, Japan***Chad Mirkin**, *Northwestern University, Evanston, IL, USA***Lei Jiang**, *Chinese Academy of Sciences, Beijing, China***Peidong Yang**, *University of California, Berkeley, CA, USA***Vivian Yam**, *The University of Hong Kong, Hong Kong*

We are very grateful and honored to cooperate with them in the development and growth of this open access platform to publish and promote the best work in the field. You will find more information about our Executive Advisory Board members on the Small Science website.

*Small Science* aims to publish all aspects of cutting‐edge research at the micro‐ and nanoscale. We welcome fundamental, applied, and especially interdisciplinary research, interesting to a very broad readership. Our first issue already illustrates the scope of our journal very well, covering topics such as porous materials, optoelectronics, nanoarchitectonics, nanophysics, energy storage, and thermoelectrics. The “small science” described in this issue has the potential to address today's societal challenges and advance to big ideas that solve the challenges of the future.

For a limited time only, we are waiving the article publication charge. Do not miss the chance to take advantage of the waiver period: submit your big ideas to us now!

We hope you enjoy our first issue, and we welcome you as new authors and readers of *Small Science*.

On behalf of the whole editorial team,

*Lu Shi* and *Ulf Scheffler*

